# Tropomyosin Receptor Kinase C Targeted Delivery of a Peptidomimetic Ligand-Photosensitizer Conjugate Induces Antitumor Immune Responses Following Photodynamic Therapy

**DOI:** 10.1038/srep37209

**Published:** 2016-11-17

**Authors:** Chin Siang Kue, Anyanee Kamkaew, Siew Hui Voon, Lik Voon Kiew, Lip Yong Chung, Kevin Burgess, Hong Boon Lee

**Affiliations:** 1 Department of Pharmacology, Faculty of Medicine, University of Malaya, 50603 Kuala Lumpur, Malaysia; 2 Department of Chemistry, Texas A & M University, Box 30012, College Station, Texas 77842, United States; 3 Department of Pharmacy, Faculty of Medicine, University of Malaya, 50603 Kuala Lumpur, Malaysia

## Abstract

Tropomyosin receptor kinase C (TrkC) targeted ligand-photosensitizer construct, IYIY-diiodo-boron-dipyrromethene (IYIY-I_2_-BODIPY) and its scrambled counterpart YIYI-I_2_-BODIPY have been prepared. IYIY-I_2_-BODIPY binds TrkC similar to neurotrophin-3 (NT-3), and NT-3 has been reported to modulate immune responses. Moreover, it could be shown that photodynamic therapy (PDT) elevates antitumor immune responses. This prompted us to investigate the immunological impacts mediated by IYIY-I_2_-BODIPY in pre- and post-PDT conditions. We demonstrated that IYIY-I_2_-BODIPY (strong response) and YIYI-I_2_-BODIPY (weak response) at 10 mg/kg, but not I_2_-BODIPY control, increased the levels of IL-2, IL-4, IL-6 and IL-17, but decreased the levels of systemic immunoregulatory mediators TGF-β, myeloid-derived suppressor cells and regulatory T-cells. Only IYIY-I_2_-BODIPY enhanced the IFN-γ^+^ and IL-17^+^ T-lymphocytes, and delayed tumor growth (~20% smaller size) in mice when administrated daily for 5 days. All those effects were observed without irradiation; when irradiated (520 nm, 100 J/cm^2^, 160 mW/cm^2^) to produce PDT effects (drug-light interval 1 h), IYIY-I_2_-BODIPY induced stronger responses. Moreover, photoirradiated IYIY-I_2_-BODIPY treated mice had high levels of effector T-cells compared to controls. Adoptive transfer of immune cells from IYIY-I_2_-BODIPY-treated survivor mice that were photoirradiated gave significantly delayed tumor growth (~40–50% smaller size) in recipient mice. IYIY-I_2_-BODIPY alone and in combination with PDT modulates the immune response in such a way that tumor growth is suppressed. Unlike immunosuppressive conventional chemotherapy, IYIY-I_2_-BODIPY can act as an immune-stimulatory chemotherapeutic agent with potential applications in clinical cancer treatment.

Conventional cancer chemotherapy is frequently associated with non-selective toxicity, treatment resistance and immune response silencing^1^,^2^. These restrictions generally lessen the effectiveness of chemotherapy. Actively targeted cancer therapies guide the agents to biomolecules (proteins, sugar or lipids) overexpressed on the cell surface, thus increasing their cellular uptake through the endocytic internalization^3^. Extensive studies have been carried out to design drug conjugates that selectively bind receptors (generally survival or metastasis biomarker in cancer) such as biotin, folate, sigma-2, carbonic anhydrase IX, glucose receptors and others^4^,^5^. The delivery agents used are generally natural ligands such as hormones, glucose derivatives, vitamins or synthetic small molecules ligands that possess similar biological functions^6^.

Our studies focus on the tropomyosin receptor kinase (Trk). These receptors are found in neurons where they regulate the neuronal cell survival and growth, proliferation, differentiation and synaptic strength and plasticity^7^, but also in neuroblastoma^8^,^9^, glioblastoma^10^, thyroid cancer^11^, melanoma^12^ and breast cancer^13^ where they impact malignancy. The expression and function of Trk subtypes are dependent on the tumor type. In neuroblastoma, TrkC expression correlates with good prognosis, but in breast, prostate and pancreatic cancers, the expression of the same Trk subtype is associated with cancer progression and metastasis^13^,^14^. Furthermore, ligands binding Trk receptors activate downstream intracellular signalling pathways that enhance tumor cell mitogenicity and survival^8^,^9^. Inhibition of Trk signaling significantly reduced tumorigenicity and invasive capability of tumor cells in *in vivo* xenograft models^13^,^15^. Several Trk receptors targeted chemotherapeutic drugs which are inhibitors of all TrkA/B/C receptors, are currently in clinical trials for treatment of solid tumors^16^.

Trk receptors and their ligands have been reported to modulate the immune system. The natural ligands of Trk receptors, neurotrophins, which include neurotrophin-3/-4 (NT-3/NT-4), brain-derived neurotrophin factor (BDNF) and neurotrophin growth factors (NGF), can function as non-cytokine mediators to modulate both innate and adaptive immune responses. Such modulations include increasing the pluripotent cytokine interleukin (IL)-6 secretion in bone marrow stromal cells^17^,^18^. In addition, neurotrophins have been reported to enhance differentiation of granulocytes (*eg* eosinophils, mast cells and basophils) during haematopoeisis^19^. In T-lymphocytes, neurotrophins regulate T cell subtypes balancing upon binding to TrkC expressing T helper (Th) 2 cell by promoting IL-4 release, which in turn blocks Th1 subtype and IFN-γ production^20^. Other than neurotrophins, TrkC was also reported to suppress transforming growth factor (TGF)-β signaling by directly binding to type II TGF-β receptor to block the association with type I receptor as well as to reduce TGF-β mediated downstream Smad2/3 phosphorylation in TrkC expressing cells^21^. Furthermore, Trk receptors are expressed in small quantities in monocytes and lymphocytes. However, despite all the above evidence that links Trk receptors to modulation of the immune system, there are currently no reports that explore Trk receptors in the context of possible strategies for immune therapy.

Photosensitizers (PS) are agents used in photodynamic therapy (PDT). In anticancer PDT, the administered PS is activated upon irradiation to generate singlet oxygen species to kill tumor cells. Diiodo boron dipyrromethene (I_2_-BODIPY) is a synthetic PS that has been reported to have high extinction coefficient and good light-to-dark toxicity ratio, fulfilling the criteria of a favorable PS^22^,^23^. However, I_2_-BODIPY has poor localization in tumor. Voon *et al*. has reported the development of nanoparticles-coated I_2_-BODIPY for passive delivery to the tumor region^24^. For an active targeting approach, we designed a TrkC receptor targeted I_2_-BODIPY construct, consisting of a synthetic TrkC ligand (Isoleucine-Tyrosine-Isoleucine-Tyrosine, IY-IY) conjugated to I_2_-BODIPY, termed as IYIY-I_2_-BODIPY (Fig. 1a). A scrambled, non-TrkC targeted ligand conjugate YIYI-I_2_-BODIPY and I_2_-BODIPY were used as controls (Fig. 1b)^25^,^26^, with and without light irradiation.

In our previous study^6^, the IYIY-ligand was found to induce some biological properties that are similar to neurotrophin-3, including internalization of the ligand-receptor complex into lysosomes, and transduction of signals that regulate neuronal cell survival and differentiation. Moreover, other researchers have shown that the resultant tumor lysate post irradiation can stimulate antigen specific immune responses to mediate short-term tumor killing and long-term tumor immunity^27^,^28^,^29^. Based on these and the previously reported immunological effects of TrkC and NT-3, we further investigated the systemic immunological impacts seen on the administration of the IYIY-I_2_-BODIPY conjugate in a preclinical murine model. Specifically, we examined the systemic modulation of cytokine levels, characterization of myeloid innate immune responses and adaptive immune T cell subtypes populations upon IYIY-I_2_-BODIPY administration in the absence of photo-activation (dark). As the I_2_-BODIPY is not active in the dark, studying the IYIY-I_2_-BODIPY construct in this setting may help to elucidate the immune-regulatory function of the IYIY ligand. This may be important, especially for PDT agents that have long drug-light interval (DLI) since the ligand in the conjugate may exert its therapeutic effect even before photo-activation of the PS. This is the first study on the immunomodulation properties and anti-tumor activity of a synthetic TrkC ligand. In addition, combination of the IYIY ligand targeting effect and simultaneous photo-activation of the I_2_-BODIPY component on immune responses was investigated.

## Results

### IYIY-I_2_-BODIPY and YIYI-I_2_-BODIPY conjugates increase pro-inflammatory cytokines IL-2, IL-4, IL-6, IL-17 and decrease immunosuppressive cytokine TGF-β in peripheral blood

In the initiation phase of the adaptive immune response, antigen-specific lymphocytes (T and B cells) are activated when they are exposed to antigen. Such activation of T-helper (Th) CD4^+^ lymphocytes causes them to differentiate into four different subtypes (Th1, Th2, Th17, regulatory T cells) depending on the availability of the different types of differentiation factors (cytokines) in the environment. The differentiated subtypes of T cells then secrete various specific cytokines. For instance, Th1 secrete IL-2, IFN-γ, TNF-α, Th2 secrete IL-4, IL-6, IL-10, Th17 secrete IL-17 and regulatory T cells (Treg) secrete TGF-β. These cytokines stimulate CD8^+^ T cells such as cytotoxic T lymphocytes (CTL), T cytotoxic (Tc)-17 cells to facilitate further adaptive immune responses. The following experiments were conducted to examine if IYIY-BODIPY modulates the cytokines secretion.

**Figure 2:**
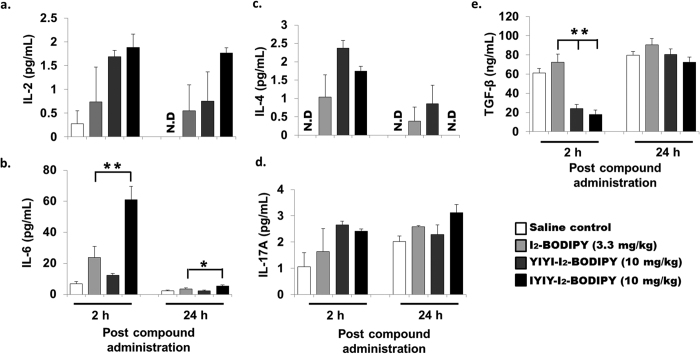
IYIY-I_2_-BODIPY and YIYI-I_2_-BODIPY conjugates increase IL-2, IL-6, IL-17 and decrease TGF-β cytokine levels. 4T1 tumor bearing mice were randomly divided into four treatment groups (saline, I_2_-BODIPY, YIYI-I_2_-BODIPY and IYIY-I_2_-BODIPY) and compounds were administrated via tail vein respectively. Mice were then sacrificed at 2 h and 24 h post compound administration. The blood plasma was extracted from these mice via cardiac puncture for cytokines analysis. Levels of cytokines (**a**) IL-2, (**b**) IL-6, (**c**) IL-4, (**d**) IL-17A and (**e**) TGF-β are shown. Data represent mean ± SEM with minimum of four mice per group. *p < 0.05, **p < 0.005 refers to comparison between I_2_-BODIPY and conjugates using one-way ANOVA (Dunnett’s test).

We first investigated the cytokine levels in TrkC + murine 4T1 breast cancer model^13^ that has been treated with IYIY-I_2_-BODIPY, YIYI-I_2_-BODIPY or I_2_-BODIPY, the latter two as controls. Blood plasma samples were collected from 4T1 tumor bearing mice via cardiac puncture at 2 h and 24 h post compound administration with no photo-activation (hereafter referred to as dark treatment) to examine early and late immune responses respectively. All treatment groups had significant increased levels of Th1 cytokine IL-2 at 2 h and 24 h (0.7–1.9 pg/mL) compared to saline (0–0.3 pg/mL; *p* < 0.05 for IYIY-I_2_-BODIPY and YIYI-I_2_-BODIPY *vs* saline at 2 h; Fig. 2a). Since IL-2 is usually produced to promote activation and proliferation of T cells^30^, the observed elevation of IL-2 suggests that investigated compounds in the dark induced T cell responses. Increases in levels of other Th1 cytokines such as IFN-γ and TNF-α were hardly significant in all treatment groups compared to the mice treated with saline control (Supplementary Figure 1a,b), suggesting that Th1-mediated inflammation was mild.

IL-6 is a set of pluripotent pro-inflammatory cytokines that regulate differentiation and functions of T lymphocytes as well as expansion of myeloid cells^31^,^32^. The levels of IL-6 were significantly elevated in IYIY-I_2_-BODIPY treated mice (approximately 7.5-, 2.6- and 4.6-fold compared to mice treated with saline control, I_2_-BODIPY and YIYI-I_2_-BODIPY respectively at 2 h; *p* < 0.05). The increase of IL-6 level did not persist, as the level at 24 h decreased even though it was still significantly higher compared to other groups (Fig. 2b).

Another cytokine, namely IL-4 was measured with 1 to 2.4 pg/mL in all mice treated with the IYIY-I_2_-BODIPY, YIYI-I_2_-BODIPY and I_2_-BODIPY at 2 h only compared to mice treated with saline (undetected, Fig. 2c), in a non-compound specific manner. At 24 h, IL-4 levels decreased in all the compound treated groups. Levels of the Th2 immunosuppressive cytokine IL-10 induced by these compounds were comparable (6–9 pg/mL) in these experiments (Supplementary Figure 1c). Together, the data indicate that the Th2 cytokines (IL-4, IL-6) were transiently elevated, whereas the Th1 response were very mild (weak TNF-α at 2 h, no change for IFN-γ). In addition, there was minor increase (1.3-fold) in the level of pro-inflammatory IL-17A which is specifically secreted by IL-17^+^ T cells, only in IYIY-I_2_-BODIPY treated group at 24 h compared to other treatment and control groups. There was no significant change in the IL-17A levels at 2 h post compound administration when compared to mice treated with I_2_-BODIPY (Fig. 2d). Furthermore, levels of immunosuppressive cytokine TGF-β was significantly suppressed only in IYIY-I_2_-BODIPY and YIYI-I_2_-BODIPY treated groups at 2 h (4.0-fold and 3.0-fold respectively, Fig. 2e) compared to mice treated with I_2_-BODIPY.

Based on the non-persistent increases of IL-4 and IL-6, as well as the declining level of IL-2 with time, we concluded that ligand-drug conjugate treated mice selectively provoke transient inflammation. Among the compounds investigated, IYIY-I_2_-BODIPY had the highest immune modulation activity. We had previously reported that YIYI-I_2_-BODIPY possessed positive but lower targeting selectivity on TrkC^+^ cells compared to IYIY-I_2_-BODIPY^26^. Therefore, it is conceivable that the YIYI ligand could elicit similar but weaker immune responses compared to IYIY, as reflected by the levels of IL-2, IL-4 and TGF-β. I_2_-BODIPY alone in the dark only caused mild immune responses albeit with statistical significance in some cases (*p* < 0.05 for IL-6 at 2 h, IL-4 at 2 h and 24 h, IL-2 at 24 h, compared to saline). This further indicated that the immunomodulation properties of the TrkC ligand-PS conjugate was mainly contributed by the TrkC ligand counterpart. In addition, the data was also in agreement with the previous study showing that YIYI-ligand had weak binding affinity on TrkC receptor^26^.

### IYIY-I_2_-BODIPY and YIYI-I_2_-BODIPY conjugates reduce populations of immunosuppressive cells

Cancer growth is normally accompanied by expansion of a heterogeneous group of immunosuppressive cells collectively known as myeloid derived suppressor cells (MDSCs). In particular, the granulocytic-MDSC (G-MDSC) subtype promotes tumor relapse by impairing the effectiveness of host immunity through inhibition of T-cell activation when primed by the tumor antigen^33^,^34^. Cancer growth is also associated with an increase in regulatory T cells (Treg) to suppress antitumor T cell responses as a method of escaping immune-surveillance. The expansion and differentiation of MDSCs and Treg cells are known to be mainly regulated by cytokines IL-6^31^,^32^ and TGF-β, respectively. Following the increase of IL-6 in IYIY-I_2_-BODIPY treated mice and decrease of TGF-β in both conjugate-treated mice, we sought to investigate changes in G-MDSCs and Treg cell populations in these animals.

**Figure 3:**
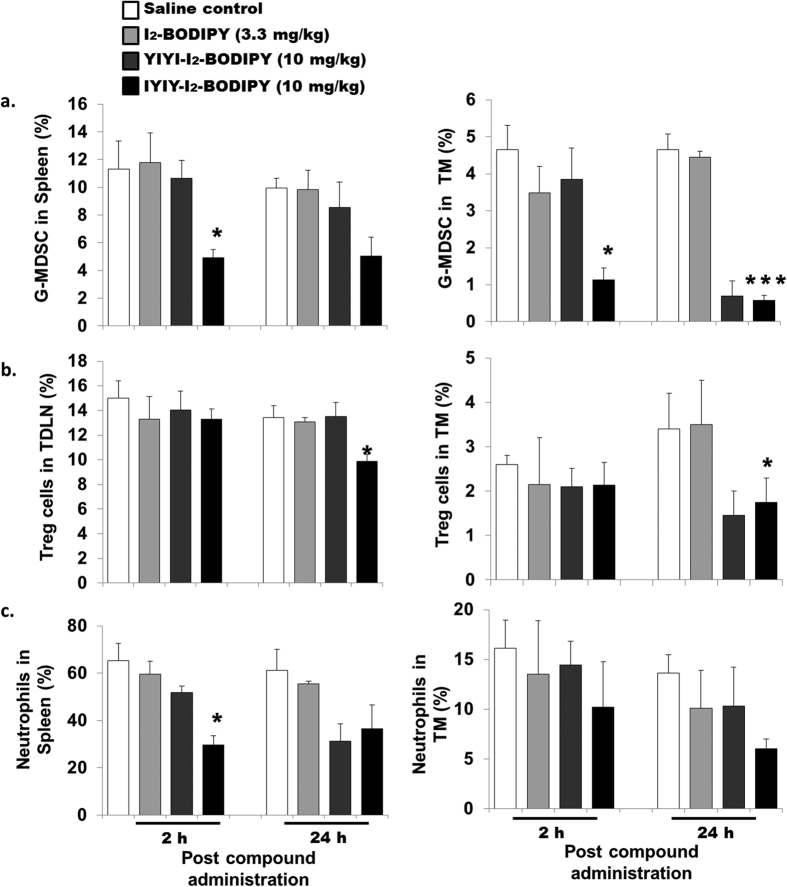
Lymphoid organs and tumor microenvironment (TM) have lowered populations of myeloid cells and Treg cells in ligand-conjugates treated mice. 4T1 tumor bearing mice were randomly divided into four treatment groups (saline, I_2_-BODIPY, YIYI-I_2_-BODIPY and IYIY-I_2_-BODIPY) and compounds were administrated via tail vein respectively. Mice were then sacrificed at 2 h and 24 h post compound administration. At scheduled time points post compound administration, mice were sacrificed, followed by isolation of TDLN, spleen and tumor tissues. Immuno-phenotyping of (**a**) G-MDSC, (**b**) Treg and (**c**) neutrophil cells was conducted using surface staining of fluorescence conjugated antibodies and quantified using flow cytometry. Data represent mean ± SEM with minimum of four mice per group. *p < 0.05, ***p < 0.001 *vs* I_2_-BODIPY using one-way ANOVA (Dunnett’s test).

IYIY-I_2_-BODIPY treated mice had significant lower level of G-MDSCs (CD11b^+^ Ly6G^+^) in spleen (4.9% ± 0.5%; *p* < 0.05) and tumor microenvironment (TM) (1.1% ± 0.3%; *p* < 0.05) at 2 h, at approximately 2-fold and 4-fold lower respectively compared to the other three control groups. Comparing the two ligated conjugates in TM, YIYI-I_2_-BODIPY treated mice showed low G-MDSC population only at 24 h, whereas IYIY-I_2_-BODIPY treated group had low G-MDSC population at 2 h and remained low up to 24 h (Fig. 3a). Similarly, in the case of Treg cells, the population in IYIY-I_2_-BODIPY mice was found to decrease by approximately 1.4-fold in TDLN at 24 h (*p* < 0.05) and 2-fold in TM at 24 h (*p* < 0.05) compared to mice treated with I_2_-BODIPY (Fig. 3b). Similar observations were made in experiments featuring YIYI-I_2_-BODIPY treated mice only at 24 h in TM. The decrease in Treg cells concurred with the depressed levels of the immunosuppressive cytokine TGF-β in both groups of ligands. Together, the data suggest that IYIY-I_2_-BODIPY was more rapid in reducing the populations of immunosuppressive G-MDSC and Treg compared to YIYI-I_2_-BODIPY, which was low only at the later time point (24 h) in TM.

Another subtype of myeloid cells, the neutrophils, was examined to confirm the suppressive activity of IYIY-I_2_-BODIPY on myeloid cell subsets. Neutrophils are defined by the expression of surface antigens CD11b and Ly6G, with the absence of the macrophage marker F4/80^35^. Similar to G-MDSC, the neutrophil population at 2 h was reduced. The reduction was 50% in spleen (*p* < 0.05) and 24% in TM (statistically insignificant) of IYIY-I_2_-BODIPY treated mice compared to mice treated with I_2_-BODIPY (Fig. 3c). In YIYI-I_2_-BODIPY treated mice, the neutrophil population was reduced by 13% at 2 h compared to mice treated with I_2_-BODIPY and at 24 h, the reduction was approximately 44% in spleen. As IL-6 is known to regulate MDSC expansion, our data suggests that the ligand-conjugates had direct impact on myeloid cells, rather than through IL-6 regulation.

### IYIY-I_2_-BODIPY enhances T-lymphocytes population with IFN-γ (Th1, CTL) and IL-17 (Th17, Tc17) phenotype

Different T-lymphocyte subtypes secrete cytokines into systemic circulation, and these cytokines especially IL-2 can affect the differentiation, expansion and survival of T-lymphocytes via autocrine or paracrine systems^36^. Consequently, the impact of ligand-drug conjugates on T-lymphocyte subtypes populations was examined.

**Figure 4:**
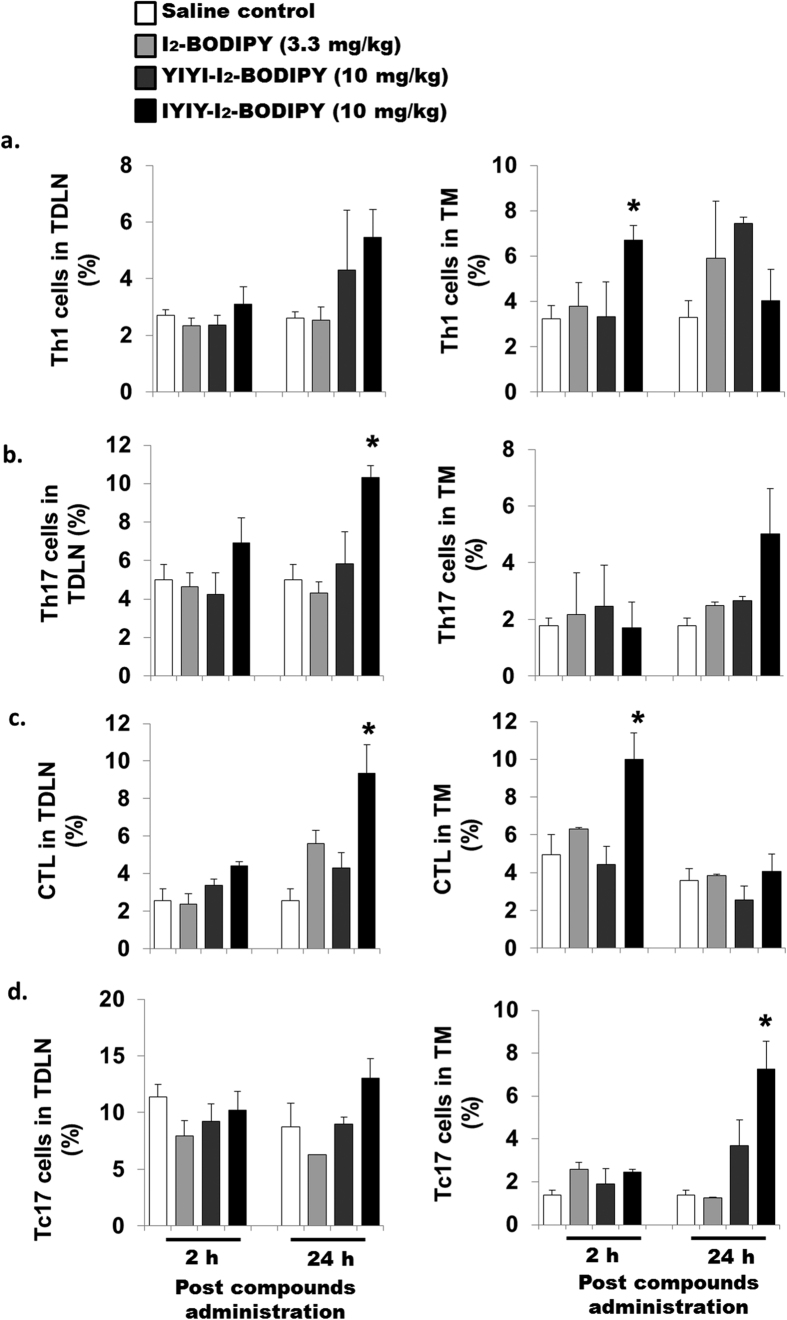
IYIY-I_2_-BODIPY increases CD4^+^ and CD8^+^ T-lymphocytes expressing IFN-γ and IL-17. 4T1 tumor bearing mice were randomly divided into four treatment groups (saline, I_2_-BODIPY, YIYI-I_2_-BODIPY and IYIY-I_2_-BODIPY) and compounds were administrated via tail vein respectively. Mice were then sacrificed at 2 h and 24 h post compound administration. TDLN and tumor tissues were isolated to generate single cells suspension. The suspension cells were activated using PMA/Ionomycin/golgi plug as described in Methods and Materials. Upon activation, CD4 marker was stained, followed by fixation and permeabilization for intracellular staining. (**a**) Th1 cells were stained with fluorescence conjugated anti-IFN-γ, (**b**) Th17 with anti-IL-17. CD8 marker was stained, follow by intracellular staining with (**c**) anti-IFN-γ (CTL) and (**d**) anti-IL-17 (Tc17). Each group of cells was then quantified using flow cytometry. Data represent mean ± SEM with minimum of four mice per group. *p < 0.05 *vs* I_2_-BODIPY using One-way ANOVA (Dunnett’s test).

Populations of CD4^+^ T helper (Th) cell subsets and antigen specific CD8^+^ T cell subsets were determined in the tumor draining lymph node (TDLN) and tumor microenvironment (TM). Th1 cell populations (CD4^+^ IFN-γ^+^) in IYIY-I_2_-BODIPY treated mice were 2-fold higher in TDLN at 24 h and in TM at 2 h (*p* < 0.05), compared to mice treated with I_2_-BODIPY. In the YIYI-I_2_-BODIPY treated group, the Th1 subset was less at 24 h in both TDLN (1.7-fold) and TM (1.3-fold) compared to mice treated with I_2_-BODIPY (Fig. 4a). Th2 (CD4^+^ IL-4^+^) populations in TDLN and TM were comparable among the treatment and control groups at 2 h. There was a moderate increase in TDLN at 24 h in IYIY-I_2_-BODIPY and YIYI-I_2_-BODIPY treated mice compared to mice treated with I_2_-BODIPY and saline. Unlike TDLN, TM had lower Th2 populations in both IYIY-I_2_-BODIPY and YIYI-I_2_-BODIPY-treated mice at 24 h compared to mice treated with I_2_-BODIPY and saline (Supplementary Figure 2).

The Th17 cell population of IYIY-I_2_-BODIPY treated group was increased compared to mice treated with I_2_-BODIPY by 2.4- and 2.0-fold at 24 h in TDLN (*p* < 0.05) and TM respectively (Fig. 4b). This observation coincided with the moderate increase of the IL-17 secretion. Unlike Th1, the increase in Th17 cell was found to occur only in IYIY-I_2_-BODIPY treated group. This is similar for IL-6 level which was also only elevated in IYIY-I_2_-BODIPY treated group, supporting that IL-6 selectively differentiates naive T cells to the Th17 subset. Taken together, the changes in Th population coincided well with the cytokines profiles in Fig. 2 and suggest that ligated conjugates increased Th1 and Th17 subsets of T cells population.

In addition, the CD8^+^ IFN-γ^+^ T cell populations (Cytotoxic T Lymphocytes, CTL) was significantly increased in IYIY-I_2_-BODIPY treated mice (approximately 2 fold in TDLN at 24 h and in TM at 2 h; *p* < 0.05) compared to all treatment groups (Fig. 4c). For CD8^+^ IL17^+^ effector T cells (Tc17), the population in TDLN was almost comparable among the treatment and control groups. Unlike TDLN, TM has higher Tc17 populations in IYIY-I_2_-BODIPY treated mice at 24 h (7.3% ± 1.3%; *p* < 0.05) and for YIYI-I_2_-BODIPY-treated mice (approximately 3.8%; *p* > 0.05) compared to mice treated with I_2_-BODIPY (1.3% ± 0.05%; Fig. 4d). This suggests that more effector T cells were generated following IYIY-I_2_-BODIPY administration compared to mice treated with I_2_-BODIPY, which then infiltrated to a high concentration at the TM (Fig. 4a–d). The elevation of effector T cells such as IFN-γ + and IL-17 + phenotypes is important for cytolytic activity and inflammation at tumor site.

**Figure 5:**
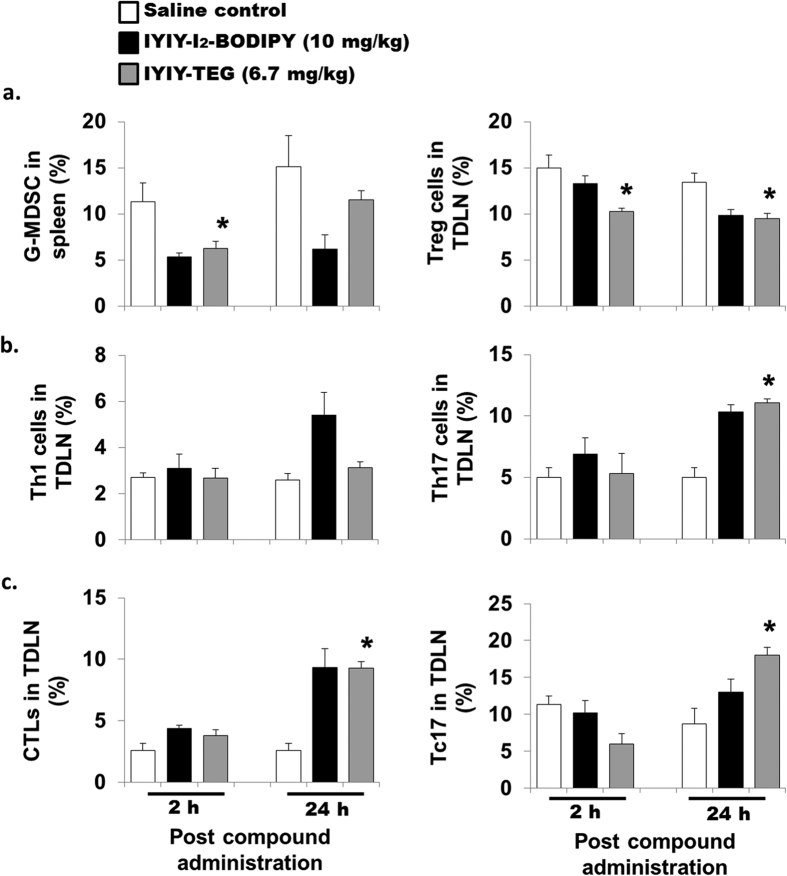
Control TrkC ligand (IYIY-TEG) has similar immunomodulatory activities as IYIY-I_2_-BODIPY. 4T1 tumor bearing mice were administrated with IYIY-TEG via tail vein (equivalent dose to IYIY-I_2_-BODIPY) and mice were sacrificed at 2 h and 24 h post administration. Lymphoid organs were harvested and populations of (**a**) G-MDSCs and Treg cells (**b**) Th1 (CD4^+^ IFN-γ^+^), Th17 (CD4^+^ IL-17^+^) and (**c**) CTLs (CD8^+^ IFN-γ^+^), Tc17 (CD8^+^ IL-17^+^) cells were quantified using flow cytometry after labeling with fluorescence conjugated antibodies. Data represent mean ± SEM with minimum of four mice per group. *p < 0.05 *vs* saline control using student’s *t-test*.

Immunomodulation properties of TrkC ligands were further confirmed when another control, IYIY-TEG (TrkC ligand without PS) reduced the immunosuppressive G-MDSC at 2 h and Treg cells (Fig. 5a), as well as increased Th17 (Fig. 5b), CTLs and Tc17 (Fig. 5c) at 24 h post administration, in the mice lymphoid organs. While no major changes were observed in the other parameters measured (G-MDSC at 24 h, Th1 cells and at 2 h time points of Th17 cells, CTLs and Tc17), these results largely mirror those produced by IYIY-I_2_-BODIPY. Based on this and the lack of immune activity from free I_2_-BODIPY as observed in Figs 2, 3 and 4, we propose that the following are direct effects of the IYIY ligand itself: (i) reduction of immunosuppressive mediators TGF-β, G-MDSC and Treg; and, (ii) elevation of adaptive immune responses with IFN-γ and IL-17 phenotypes. However, detailed mechanisms on how the ligands induced immune responses require further investigation.

### TrkC blocking antibodies reverse the TrkC conjugates-mediated myeloid cell reduction

**Figure 6:**
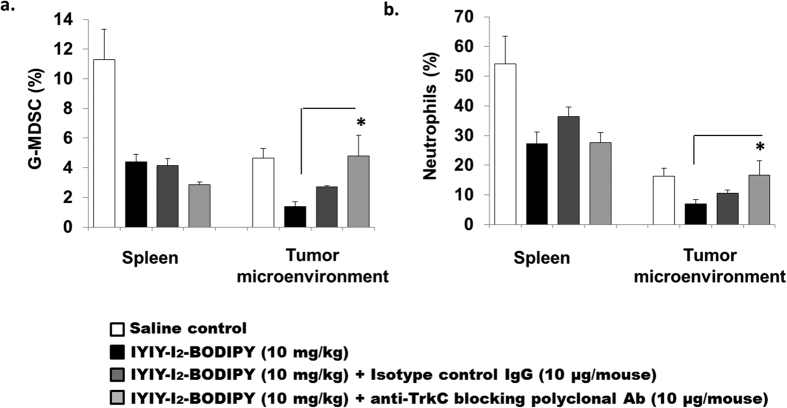
IYIY-I_2_-BODIPY mediated myeloid cells reduction is TrkC dependent. 4T1 tumor bearing mouse were randomly divided into four groups (saline, IYIY-I_2_-BODIPY, IYIY-I_2_-BODIPY + 10 μg/mouse isotype control IgG, IYIY-I_2_-BODIPY + 10 μg/mouse anti-TrkC blocking polyclonal antibodies). Compound, alone or in combination with antibodies as described was administrated into mice via tail vein, respectively. Mice were sacrificed 2 h after compounds administration. Percentages of (**a**) G-MDSCs and (**b**) neutrophils in spleen and TM were quantified using flow cytometry after cell surface staining. Data represent mean ± SEM of four mice. *p < 0.05 *vs* IYIY-I_2_-BODIPY was analyzed using student’s *t-test*.

To address whether ligated conjugates induced immune modulations were TrkC dependent *in vivo*, an experiment to co-inject either TrkC polyclonal antibodies or isotype control Immunoglobulin-G (IgG) with IYIY-I_2_-BODIPY via tail vein was performed. Due to the high immunomodulation activity of IYIY-I_2_-BODIPY, it was selected to be co-administered with antibodies. Co-injection with 10 μg/mouse of TrkC blocking antibodies reversed the IYIY-I_2_-BODIPY-mediated G-MDSC (*p* < 0.05) and neutrophil suppression (*p* < 0.05) in TM to levels similar to those in control saline, but this reversed activity was not observed to the same extent in the mice that received the isotype IgG control (Fig. 6). Reversal was not observed in the spleen G-MDSC and neutrophil levels, suggesting that the blocking effect of antibodies was local and not systemic. The reason of no reversal in spleen is unknown, but the overall result from the blocking experiment suggests that IYIY-I_2_-BODIPY mediated immunomodulation occurred in a TrkC dependent manner.

### IYIY-I_2_-BODIPY promotes mild and transient delays in tumor growth

**Figure 7:**
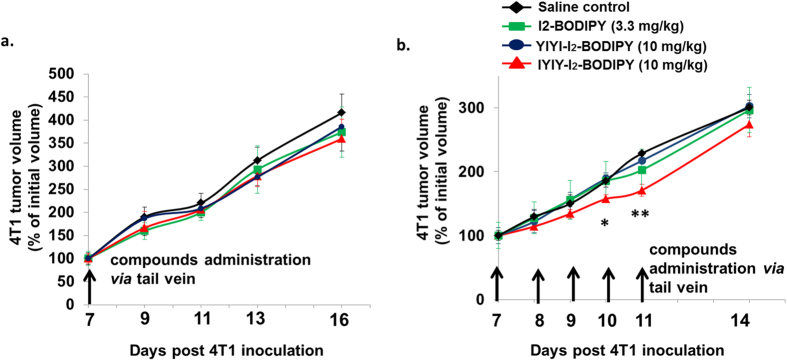
Multiple boli *i.v.* administration of IYIY-I_2_-BODIPY delays tumor growth. At 7 days post 4T1 inoculation, the mice were randomly divided into 4 groups. (**a**) For single bolus *i.v.* administration, the mice were injected with compounds (saline, I2-BODIPY, YIYI-I_2_-BODIPY and IYIY-I_2_-BODIPY) at dosages as shown. (**b**) For multiple boli *i.v.* administration, mice were injected with compounds at a regime of once a day for 5 days consecutively. Tumor growth was monitored and data represent mean percentage of initial volume ± SEM of n = 6. *p < 0.05 *vs* control saline, **p < 0.05 *vs* all three control groups using one-way ANOVA (Dunnett’s test).

Up to now, we have shown that the ligated conjugates inhibited immuno-suppressive mediators and increased adaptive immune responses in breast tumor bearing mice. Next, we examined whether treatment with the conjugates caused delays in tumor growth in the dark. The experiment involved single bolus injections of 10 mg/kg of all three compounds via tail vein, and recording of tumor volume three times per week. The data shown in Fig. 7 was quantified in comparison to the initial volume of the respective tumors to eliminate tumor-to-tumor variation. As illustrated in Fig. 7a, single bolus injection had no effect on delaying tumor growth, as the tumor sizes were comparable with saline control in all treated groups. However, multiple injections of all three compounds with a regime of 10 mg/kg every day for five consecutive days resulted in marginal delay in tumor growth only after IYIY-I_2_-BODIPY administration at the first three days following treatment (days 7–9 after tumor cells inoculation), and significant delay at days 10 (~16%) and 11 (~20%) post tumor cells injection compared to all controls (Fig. 7b). The growth delay was transient, as the tumor sizes became comparable when the administration of IYIY-I_2_-BODIPY was stopped. Even though the delay in tumor growth was statistically significant, the effect may not be strong or prolonged enough for clinical relevance. In addition, as only IYIY-I_2_-BODIPY but not YIYI-I_2_-BODIPY transiently delayed tumor growth, as well as elicited strong immune responses, our data suggest that tumor control was contributed by immune modulation.

### Combination effect of conjugates and PDT in adaptive immunity

Adaptive immunity is highly specific to the antigens presented to the immune cells and generally provides long lasting protection to the host. In cancer, adaptive immune cells such as CD4^+^ T helper cells and CD8^+^ T cells are activated through presentation of tumor antigen in Major Histocompatibility Complex (MHC)-Class II and Class I by antigen presenting cells, respectively. Activated T-lymphocytes will undergo differentiation to various subtypes depending on the cytokines milieu present. In photodynamic therapy (PDT), antigen specific adaptive immune responses are activated by release of large quantities of tumor antigens by the damaged tumor tissues^29^,^37^.

Data obtained until this point suggest that the conjugate-ligands had immuno-stimulatory properties, hence we hypothesized that photo-activation of the I_2_-BODIPY photosensitizer counterpart of IYIY-I_2_-BODIPY and YIYI-I_2_-BODIPY (hereafter referred to as light treatment) following the administration of the conjugates into the mice may induce stronger adaptive immune responses. To explore this idea, tumor bearing mice were administrated with the IYIY-I_2_-BODIPY, YIYI-I_2_-BODIPY and I_2_-BODIPY (10 mg/kg equivalent dose) and illuminated with 100 J/cm^2^ of light after a drug-light interval of 1 h^26^. Mice were then sacrificed 2 h and 24 h after PDT.

**Figure 8:**
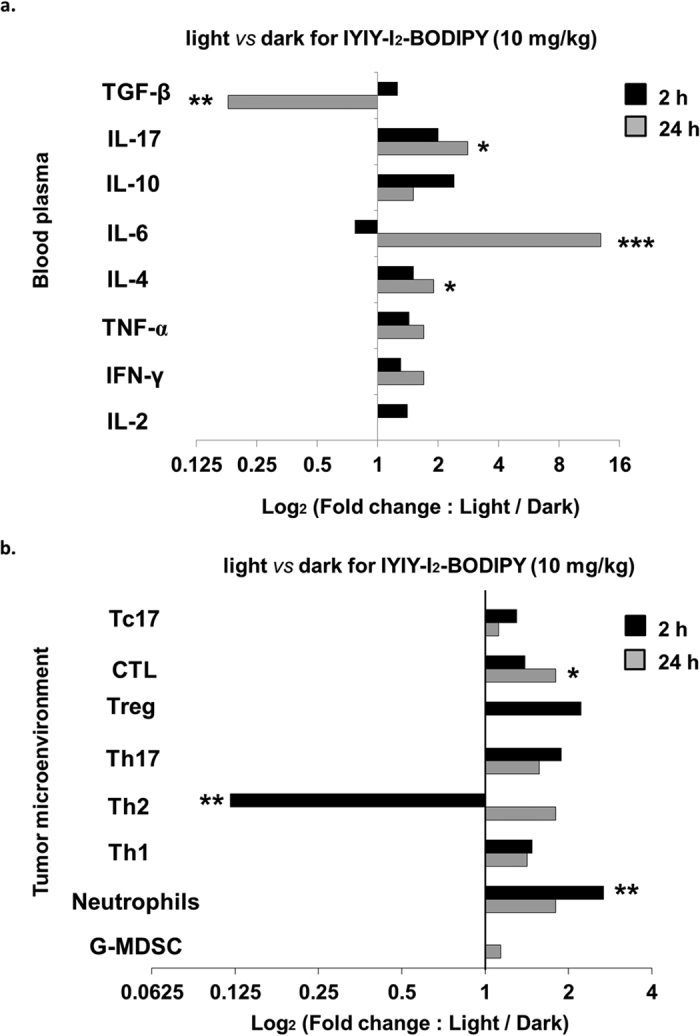
IYIY-I_2_-BODIPY combined with PDT induces stronger adaptive immunity. 4T1 tumor bearing mice were treated with respective compounds via tail vain. Mice were kept in dark for 1 h, anaesthetized and irradiated with 100 J/cm^2^ of light at a fluence rate of 160 mW/cm^2^. Mice were then sacrificed at 2 h and 24 h post-PDT. Cytokines and immune cells quantification were examined based on the methods explained in M&M. Graph of fold changes in (**a**) blood cytokines and (**b**) immune cells populations in TM of the light (Supplementary Figure 3) *versus* dark (Figs 2, 3 and 4) for 2 h and 24 h in IYIY-I_2_-BODIPY treated group. Fold changes for each sample in IYIY-I_2_-BODIPY treated group was obtained by dividing the percentage in the light over the percentage in the dark. *p < 0.05, ***p < 0.001 *vs* IYIY-I_2_-BODIPY in the dark using student’s *t-test*.

Interestingly, most of the immune modulation responses observed in the dark for the ligated conjugates were also present in the light treatment. The data for IYIY-I_2_-BODIPY, YIYI-I_2_-BODIPY and I_2_-BODIPY-induced immune modulation following PDT are illustrated in Supplementary Figure 3 (systemic cytokines level) and Supplementary Figure 4 (immune cell populations in tumor microenvironment). For ease of comparison, the data for IYIY-I_2_-BODIPY (highest immunomodulatory activity) treated mice has been re-tabulated in terms of fold changes in the blood cytokines (Fig. 8a) and immune cell populations in tumor microenvironment (Fig. 8b) in light treatment compared to those in dark, at 2 and 24 h post-PDT treatment. Among the cytokines investigated, the biggest fold changes were: (i) 5.5-fold down-regulation of TGF-β and (ii) 13-fold up-regulation of IL-6, both at 24 h. High TGF-β suppression is similar to the previously reported effect of ligand-activated TrkC receptor in inhibiting TGF-β signaling^21^, whereas the elevation of IL-6 might be due to the effect of ligand in promoting TrkC-induced IL-6 secretion^17^,^18^. IL-17A was 2.8-fold increased in the light compared to in the dark at 24 h post-PDT, further suggesting that the high IL-6 level induced by IYIY-I_2_-BODIPY had facilitated IL-17 + T-cell differentiation and IL-17 secretion. The rest of the cytokines investigated had fold changes ranging from 1.25 to 2.4 (Fig. 8a).

For immune cell populations in the tumor microenvironment (Fig. 8b), Th2 cell population was reduced in the light at 2 h post-PDT compared to in the dark. The decrease of the Th2 cells post-PDT and increase of the Th1 cell population are in line with the known subtype balancing of Th1/Th2 cells, where activation of either cell type can down-regulate the other^38^. Unlike Th2, the other immune cell types investigated in IYIY-I_2_-BODIPY treated group were in increasing trend at 2 h and 24 h post-PDT compared to in the dark. The highest increase in fold change was observed in neutrophils (2.8-fold; Fig. 8b), suggesting that IYIY-I_2_-BODIPY recruited more neutrophils to TM post-light treatment.

Altogether, our data suggest that photoactivation of I_2_-BODIPY counterpart combined with the immune response of the IYIY ligand in the IYIY-I_2_-BODIPY conjugate to enhance levels of inflammatory cytokines IL-6 and IL-17, T-lymphocyte with phenotype of IFN-γ^+^ and IL-17^+^, and inflammatory cell neutrophils. This data may also explain the superior antitumor activity that was observed for PDT when using IYIY-I_2_-BODIPY with PDT^26^.

### PDT increases effector T cells in IYIY-I_2_-BODIPY treated mice

**Figure 9:**
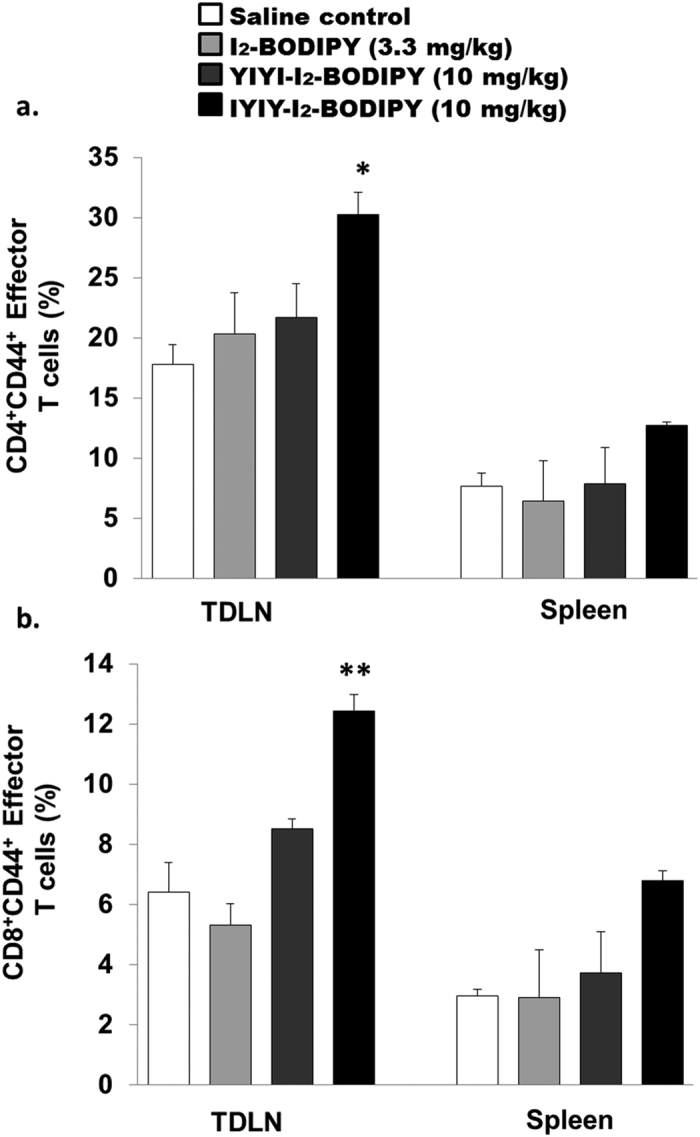
IYIY-I_2_-BODIPY treated mice have high CD4^ + ^and CD8^ + ^effector T cells in TDLN and spleen at 20 days post-PDT. PDT was conducted at 100 J/cm^2^ based on four groups as indicated above. At 20 days post-PDT, mice were sacrificed for quantification of (**a**) CD4^+^ and (**b**) CD8^+^ effector T cells in TDLN and spleen that expressed CD44 surface antigen using flow cytometry. Data represent mean ± SEM of three mice for each group. *p < 0.005; **p < 0.005 *vs* three control groups using one-way ANOVA (Dunnett’s test).

Effective PDT of cancer would induce antitumor immunity through inflammation and adaptive immune responses^39^,^40^, which could further lead to the development of effector or memory T cells against the tumor^29^. Previously we have shown that 71% of IYIY-I_2_-BODIPY treated mice were cured from tumor post PDT^26^. We sought to investigate whether IYIY-I_2_-BODIPY treated mice have increased CD4^+^ and CD8^+^ effector T-cell populations post PDT. Compounds treated mice underwent PDT and at 20 days post-PDT, TDLN and spleen were isolated and quantified for effector T cells using CD44 as a surface marker. As expected, IYIY-I_2_-BODIPY treated mice had significant high CD4^+^ (30.3 ± 1.8%, Fig. 9a) and CD8^+^ (12.4 ± 0.5%, Fig. 9b) effector T cells in TDLN compared to the other treatment and saline controls (~17–21% for CD4^+^, ~5–8% for CD8^+^), and only moderate increase in spleen (~13% CD4^+^; ~7% CD8^+^) compared to other groups (~6–8% CD4^+^; 3–4% CD8^+^). However, no increase in effector T cells was observed in YIYI-I_2_-BODIPY treated mice. This implies that IYIY-I_2_-BODIPY was a more effective PDT agent in inducing antitumor effector T cells than the YIYI conjugate.

### Survivor mice ignite antitumor activity by delaying growth of aggressive 4T1 tumor

**Figure 10:**
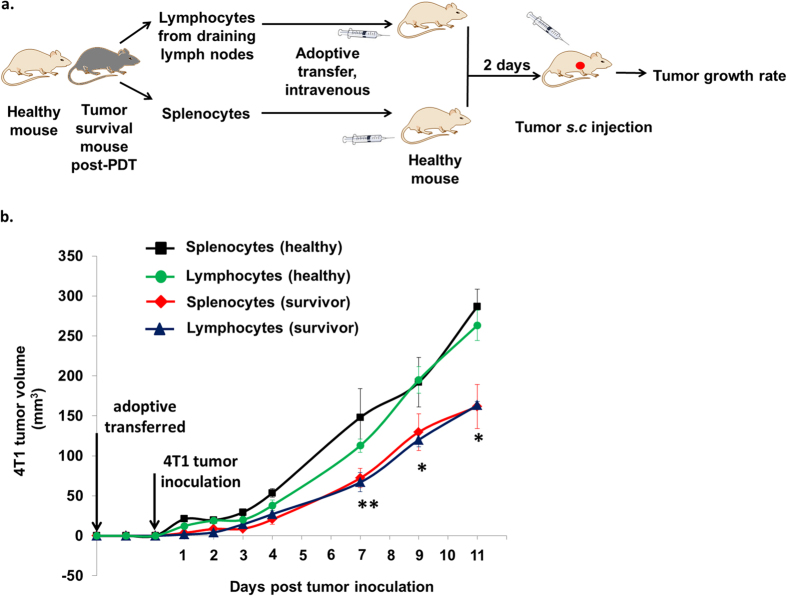
Adoptive transfer of immune cells in IYIY-I_2_-BODIPY treated survivor mice delayed tumor growth. (**a**) The schematic diagram showed the work flow for antitumor immunity studies via adoptive transfer. (**b**) 4T1 tumor volume in four groups of mice is indicated above. Data represent mean ± SEM of 5 mice. *p < 0.05, **p < 0.005 *vs* respective control (healthy) using student’s *t-test*.

**Figure 11:**
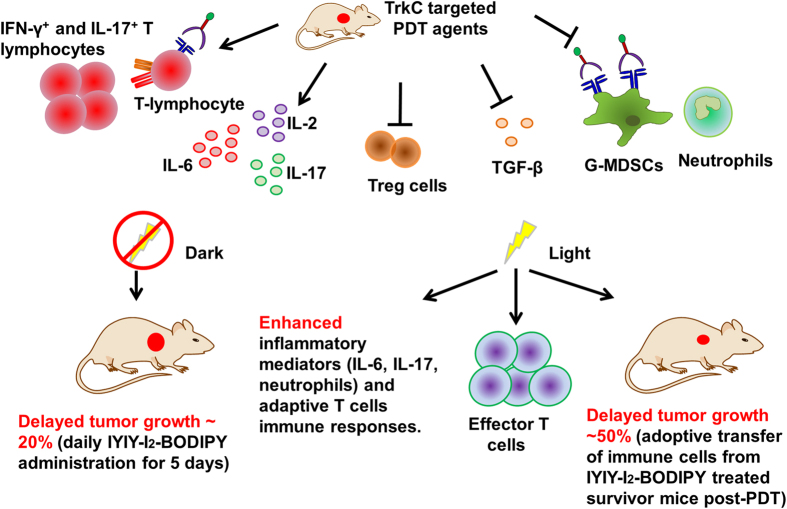
Summary of IYIY-I_2_-BODIPY mediated immune modulations in mouse 4T1 breast tumor model.

Lastly, we examined whether the IYIY-I_2_-BODIPY treated survivor mice post-PDT possess long term immunity against aggressive 4T1 tumor cells. In this study, IYIY-I_2_-BODIPY treated mice had high survival rate (60% of mice survived for up to 60 days), which was consistent with our previous study (71% of mice survived for up to 90 days)^26^. The splenocytes and lymphocytes (suspension cells from TDLN) of healthy survivor mice were isolated and adoptively transferred into syngeneic healthy recipient mice via tail vein. Recipient mice were challenged by subcutaneous injection of 4T1 tumor cells at 2 days post adoptive transfer as described in Fig. 10a. Interestingly, tumor growth delay was observed in mice receiving splenocytes and lymphocytes from survivor mice, compared to controls (splenocytes and lymphocytes from healthy mice). In brief, the delay in tumor growth was transient at days one to four post tumor inoculation and became significant (~50%) in both splenocytes and lymphocytes recipient mice at 7 days post inoculation (*p* < 0.005, Fig. 10b). The delay in tumor growth continued, with approximately 40% smaller tumors sizes in mice receiving immune cells from survivor mice at both 9 and 11 days post inoculation compared to mice with cells from healthy donors. Unfortunately, we failed to see full immunity in the mice after adoptive transfer, probably due to the highly aggressive and rapid proliferative nature of 4T1 tumor cells and failure in sorting of CD44^+^ T cells for adoptive transfer. However, the delayed tumor growth was significant as compared to controls, suggesting the presence of anti-tumor immune effector or memory T cells. Taken together, the higher survivor rate previously observed in IYIY-I_2_-BODIPY PDT treated mice compared to the other controls^26^ might have been due to the enhanced effector or memory T cells caused by the combined TrkC and PDT therapy. A summary of results of IYIY-I_2_-BODIPY-mediated immune modulation is shown in Fig. 11.

## Discussion

We examined the immunological impacts of a TrkC-targeted PDT agent upon compound administration in a TrkC + 4T1 breast tumor model. We observed that the TrkC targeted PDT conjugates in the dark transiently increased cytokines IL-2, IL-4, IL-6 and IL-17, and inhibited immunosuppressive mediators such as TGF-β cytokine, G-MDSC, Treg cells and inflammatory neutrophils. In addition, we found increases in the CD4^+^ and CD8^+^ antitumor responses with IFN-γ and IL-17 phenotypes, and growth delay of aggressive 4T1 tumors in mice. While IYIY-BODIPY in the dark activated mild immune reactions and decreased tumor growth to some extent, the assault by reactive oxygen species upon PDT accentuated those effects observed in dark. Upon PDT, the targeted conjugate-treated mice also had higher levels of IL-6, IL-17, CTL, effector T cells and lower level of TGF-β, as well as a delay in tumor growth through adoptive transfer of immune cells. In contrary to the dark, the level of neutrophils was enhanced in the conjugate PDT treated mice. To the best of our knowledge, this is the first report of antitumor immune responses elicited by a synthetic ligand designed primarily to actively directly a therapeutic agent to cancer.

Throughout the study, both in dark and light treatments, the scrambled control YIYI-I_2_-BODIPY showed similar, but weaker immune-modulation properties in Th1, CTL, TGF-β, Treg, MDSC than IYIY-I_2_-BODIPY. This might be due to the isomeric characteristics of scrambled ligands (reversed order of amino acids), leading to similar, albeit weaker binding affinity to the targeted receptor compared to the unscrambled ligands^41^. Another possibility is that the YIYI-I_2_-BODIPY scrambled control adopts a bioactive conformation that binds to other unknown receptors to also modulate immune response.

IYIY-I_2_-BODIPY induced IL-6 secretion and reduced G-MDSCs both in dark and light treatments. The increase of IL-6 by IYIY-I_2_-BODIPY was the same as other studies where neurotrophins binding to Trk receptor increased IL-6 secretion in bone marrow stromal cells^17^,^18^. The concurrent reduction in G-MDSCs however is in contrast as in other studies that reported correlative increases in IL-6 with MDSC expansion and inflammation^31^,^32^,^42^. It is possible that the TrkC ligand mediated MDSC suppression was independent of IL-6, but through direct binding to myeloid cells, since blocking with TrkC antibodies could reverse the IYIY-I_2_-BODIPY-mediated MDSC suppression. Another explanation for the mechanism of MDSC suppression may involve transcription factor STAT-3 which is an essential mediator for regulating myeloid progenitor proliferations^43^, as well as a downstream mediator of Trk signaling^44^.

Studies have revealed that the balance of Treg and Th17 differentiation is regulated by the relative abundance of differentiation factors TGF-β and IL-6. A high level of TGF-β generally promotes Treg differentiation and inhibits Th17^45^, whereas a high IL-6 level stimulates Th17 differentiation and antagonizes Treg formation^46^. Our findings on the increase of Th17 upon IYIY-I_2_-BODIPY administration both in dark and light treatments were in concordance with the latter scenario. Th17 cells secrete IL-17 cytokine that is known to possess both protumor and antitumor functions^47^. The IYIY-BODIPY mediated IL-17^+^ cells in this study probably functioned in an antitumor way based on (a) the observed delay in tumor growth post IYIY-I_2_-BODIPY administration, (b) the reduction of TGF-β which is known to block the production of vascular endothelial growth factor (VEGF, a potent angiogenic factor)^48^,^49^ and, (c) the increase in Th1 and CTL cell populations which have been reported in the presence of IL-17^50^,^51^.

TGF-β signaling has dual functions in cancer: (i) as a tumor suppressor to mediate growth arrest and apoptosis in cancer cells (antitumor)^52^,^53^, and (ii) as a potent immunosuppressive agent to suppress both innate and adaptive immune responses comprising of CD4^+^ effector T cells (Th1 and Th2), CD8^+^ cytotoxic T cells (CTLs), NK cells and antigen presenting cells, as well as to stimulate the generation of regulatory T cells which inhibit effector T cell functions (protumor)^54^. Jin and co-workers reported TrkC and its tyrosine kinase activity mediated tumorigenesis (protumor) through suppression of the tumor suppressor TGF-β signaling^21^, which is different from our study which showed conjugate-activated TrkC administered in dark inhibited TGF-β production and delayed tumorigenesis (antitumor). The discrepancy may be due to the dual function of TGF-β both as a pro- and an anti-tumor mediator^54^,^55^. To explain the antitumor role of TGF-β in our study, the binding of TrkC by the ligated conjugates might have activated the tyrosine kinase activity of TrkC and subsequently blocked the TGF-β signalling and inhibited TGF-β production at an early time point, which in turn reduced the Treg cells in a late time point to overall delay the tumor growth.

PDT can lead to systemic induction of IL-6^56^,^57^, and this cytokine has been studied to possess dual functions, either in enhancing PDT efficacy^58^,^59^ or inhibiting PDT mediated antitumor immunity^60^ via regulation of apoptotic proteins. Our PDT results in this study suggest that IL-6 was an antitumor factor, likely by enhancing PDT efficacy via upregulation of Th17 and Tc17 cells. This is contrary to the findings by Brackett *et al*.^60^, where they reported IL-6 attenuated PDT mediated antitumor immune memory in an IL-6 knockout mouse model implanted with 4T1 breast mammary tumor. The differences in findings might due to the different drugs administered (the ligand in our conjugate has additional immunomodulation properties) or the extreme difference in total systemic IL-6 level in different genetic background of mice (IL-6 knockout *vs* wild type). Overall, the role of IL-6 in PDT mediated immune responses may depend on the severity of inflammation and its action in mediating the balance between differentiation of Th17 and Treg cells.

IYIY-I_2_-BODIPY treated mice were shown here to increase recruitment of neutrophils to tumor tissues post-PDT, but this was not observed in both YIYI-I_2_-BODIPY and I_2_-BODIPY treated animals in both dark and light treatments. This can be explained by the good antitumor efficacy of IYIY-I_2_-BODIPY in causing tumor damage^26^, leading to the local accumulation of inflammatory cells such as neutrophils (Fig. 8b), as well as effector T cells (Fig. 9). This is concordant with de Vree *et al*.^40^, who proposed that high PDT efficacy is associated with increased population of neutrophils in systemic circulation.

In conclusion, stimulation of inflammatory IL-6, IL-17 cytokines and inhibition of immunosuppressive mediators are potential contributors of the immune stimulatory effects of the IYIY-ligand. This is different from other small molecules used in active targeting such as folate and estrogen which enhance the activity of immunoregulatory T cells - useful for treating autoimmune and inflammatory diseases^61^,^62^. In addition, the IYIY-I_2_-BODIPY conjugate has demonstrated potential as a therapeutic agent for cancer treatment by selectively destroying the TrkC + tumor cells in mice upon PDT with high post-treatment survival rate^26^, and at the same time stimulating antitumor immunity in the host. This suggests that TrkC targeted delivery, as demonstrated in the IYIY-I_2_-BODIPY conjugate can improve cancer treatment for patients. Further studies employing a modified BODIPY with an absorption maximum at higher wavelengths for deeper light penetration during therapeutic intervention are underway.

## Materials and Methods

### Compounds

TrkC targeted IYIY-I_2_-BODIPY (Isoleucine-Tyrosine-Isoleucine-Tyrosine, IYIY conjugated I_2_-BODIPY), non-TrkC targeted scrambled control YIYI-I_2_-BODIPY (Tyrosine-Isoleucine-Tyrosine-Isoleucine, YIYI conjugated I_2_-BODIPY), free photosensitizer I_2_-BODIPY and TrkC targeted ligand IYIY-TEG (Isoleucine-Tyrosine-Isoleucine-Tyrosine conjugated to triethylene glycol (TEG) without I_2_-BODIPY) were synthesized as previously^25^.

### Animal model

Female 8–10 week old, wild type BALB/c mice were purchased from Taconic and InVivos Pte Ltd, Singapore and maintained in the AAALAC accredited satellite animal facility at the Department of Pharmacology, Faculty OF Medicine, University of Malaya. All animal experiments were performed according to protocols approved by the Faculty of Medicine Institutional Animal Care and Use Committee, University of Malaya (FOM IACUC). Ethics approval numbers: 2013-05-07/PHA/R/KLV and 20150303/PHAR/R/KCS.

### Tumor model development

The fur of the BALB/C mouse was shaved and murine breast carcinoma 4T1 cell line (ATCC) at a density of 5 × 10^5^ cells in 0.1 mL of RPMI medium was orthotopically injected into the mammary fat pad of the mice. The mice were monitored for tumor development every day and were then randomly divided into groups for compounds administration when the size reached around 60–80 mm^3^.

### Compounds administration

IYIY-I_2_-BODIPY, YIYI-I_2_-BODIPY at 10 mg/kg of body weight (consist 3.3 mg equivalent/kg of I_2_-BODIPY and 6.7 mg equivalent/kg of IYIY-TEG) and 3.3 mg/kg of I_2_-BODIPY were dissolved respectively in a cocktail of 2.5% ethanol and 2.5% CremophoreEL in saline. The mixture was then further dissolved using saline to a volume of 0.2 mL and intravenously administrated to tumor bearing mice via tail vein. The mice were then kept in an environment away from bright light for 2 h and 24 h (n = 5 per treatment group for every time point).

### Blood sampling

At 2 h (represented innate immune response) and 24 h (represented onset of adaptive immune response) time points post compounds administration, mice from each group (n = 5 per time point) were anaesthetized with anaesthesia cocktail (90 mg/kg of ketamine and 10 mg/kg of xylazine). 0.5 mL of blood was withdrawn via cardiac puncture after the onset of anaesthesia.

### Flow cytometry quantification of plasma cytokines

The withdrawn blood was centrifuged at 5000 rpm for 10 minutes to separate the plasma and peripheral blood mononuclear cells. Cytokine levels in blood plasma were then determined using a BD Cytometric Bead Array (CBA) Mouse Th1/Th2/Th17 Cytokine Kit (BD Biosciences, San Jose, CA) according to manufacturer’s instructions. Cytokines IL-2, IL-4, IL-6, IFN-γ, TNF-α, IL-17A and IL-10 were quantified using FACSCanto II (BD Biosciences) and analysed using FCAP array software (BD Biosciences). TGF-β cytokine in blood plasma was quantified using Human/Mouse TGF-β1 sandwich ELISA (eBiosciences, San Diego, CA) based on manufacturer’s instructions.

### Cell isolation from lymphoid organs and tumor tissues

After the blood sampling at each indicated time points, mice (n = 5 per time point) were sacrificed and tumor draining lymph nodes (TDLN), spleen and tumor tissues were harvested. Single cell suspensions from TDLN and spleen were obtained by mincing the organs using frosted glass slides, followed by red blood cell lysis using red blood cell lysis buffer. Single cell suspensions from tumor tissues were obtained by mincing tumor tissue with frosted glass slides, followed by digestion with 0.5 mg/mL of collagenase type XI (Sigma Aldrich, St Louis, MO) for 30 minutes in a 37 ^o^C shaker at 200 rpm. The cell suspensions were then filtered to eliminate the debris. All the single cell suspensions from TDLN and tumor tissues were stimulated for 4 h with 50 ng/mL of Phorbol 12-Myristate 13-Acetate (PMA), 1 μg/mL of Ionomycin (Sigma Aldrich, St Louis, MO) and 3 μg/mL of brefeldin A (BD Bioscience, San Jose, CA)^63^.

### Staining and flow cytometry quantification of immune cells

The stimulated single cell suspensions from tumor tissues, TDLN and spleen were washed with PBS, and resuspended (1 × 10^6^ cells) in FACS washing buffer (0.5% FBS and 0.05% sodium azide in PBS). The cells were then stained with a panel of fluorochromes conjugated monoclonal antibodies purchased from BD Pharmingen to detect specific surface antigen that are FITC-anti mouse CD11b (553310), PE-anti mouse Ly6G (551461), APC-anti mouse F4/80 (17-4801), FITC-anti mouse CD4 (553729), PE-anti mouse CD25 (553075), FITC anti mouse CD8 (553031). The cells were fixed and permeabilized using Cytofix/Cytoperm kit (BD Biosciences, San Jose, CA) according to the manufacturer’s instructions. The surface antigens stained cells were then labelled for intracellular cytokine using PE-anti-mouse IFN-γ (554412), PerCP-Cy5.5-anti-mouse IL-4 (560700), APC-anti-mouse IL-17 (17-7177), and APC-anti-mouse FoxP3 (17-5773) with different fluorochrome combination to avoid overlap. We quantified the cells of interest based on surface markers, Th1 (CD4^+^ IFN-γ^+^), Th2 (CD4^+^ IL-4^+^), Th17 (CD4^+^ IL-17^+^), Treg (CD4^+^ CD25^+^ FoxP3^+^), CTL (CD8^+^ IFN-γ^+^), Tc-17 (CD8^+^ IL-17^+^), granulocytic-MDSC (CD11b^+^ Ly6G^+^, neutrophils (CD11b^+^ Ly6G^+^ F4/80^−^). Effector T cells were stained using PE-Cy5 rat anti-mouse CD44 (553135) for CD4^+^ CD44^high^ and CD8^+^ CD44^high^ populations. A total of 10,000 cells were analysed for every experiment. The stained cells was analysed in a FACS Canto-II Flow Cytometry (BD Biosciences, San Jose, CA). The data were analysed using Cell Quest software (BD Biosciences, San Jose, CA).

### *In vivo* TrkC blocking studies

For TrkC blocking study, the tumor bearing mice (n = 4 per group) were treated with IYIY-I_2_-BODIPY (10 mg/kg) with or without mouse TrkC antibody (R&D systems, Minneapolis, MN) at a dosage of 10 μg/mouse via tail vein and kept away from bright light as a non-irradiated study. Tumor bearing mice receiving isotype control Immunoglobulin G (R&D systems, Minneapolis, MN) at dosage of 10 μg/mouse (n = 4) was used as an antibody control in this study. At 2 h post IYIY-I_2_-BODIPY and antibodies administration, the mice were sacrificed and tumor tissues, spleen and TDLN were harvested, stained with respective fluorescence conjugates antibodies and phenotyped for granulocytic-MDSC, neutrophils, CD4^+^ and CD8^+^ T cell subtypes using flow cytometry.

### Photodynamic therapy (PDT) in mice

For irradiation, IYIY-I_2_-BODIPY (10 mg/kg), YIYI-I_2_-BODIPY (10 mg/kg), I_2_-BODIPY (3.3 mg/kg) and saline were given respectively to the mice (n = 5 per group). The mice were kept away from bright light post compound administration for 1 h. Thereafter, anaesthesia cocktail of 90 mg/kg of ketamine and 10 mg/kg of xylazine was given to the mice. Upon the onset of anaesthesia, PDT was performed using a Lumacare LC-122A fiber optic light delivery system (standard fiber optic probe model LUM V, 400–700 nm, Lumacare Medical Group, Newport Beach, CA, USA, with a 500/585 nm bandpass filter from Omega Optical) to emit light at 530 nm. A 4 mm thick glass slide was used as a barrier to avoid direct photothermal effect on tumor. The illuminating spot was positioned at the tumor and the surrounding was covered using a black cloth to avoid PDT effect on non-tumor region of body. PDT was conducted at 100 J/cm^2^ with a fluence rate of 160 mW/cm^2^, for 10 minutes. The irradiated mice were then sacrificed for blood and lymphoid organ isolation at 2 h and 24 h post-PDT. The methods of samples processing in irradiated mice were same as methods described above (blood sampling, flow cytometry quantification of plasma cytokines, cell isolation from lymphoid organs and tumor tissues, staining and flow cytometry quantification of immune cells).

### Adoptive transfer for antitumor immunity

For adoptive transfer studies, spleen and TDLN from healthy mice (n = 5) and the survivor mice (tumor free 60 days post PDT) in IYIY-I_2_-BODIPY treated group of mice (n = 5) were harvested. Splenocytes at a density of 2 × 10^7^ cells/0.2 mL/mouse and lymphocytes from TDLN at a density of 1.5 × 10^7^ cells/0.2 mL/mouse from each mouse were injected into different recipients of healthy syngeneic mice (n = 5) via the tail vein. After 2 days of adoptive transfer, the recipient mice were inoculated subcutaneously with 4T1 tumor cells at a density of 5 × 10^5^ cells/0.1 mL/mouse. The tumor growth in the mice that received survivor splenocytes and lymphocytes was monitored and compared with the mice that received splenocytes and lymphocytes from the healthy mice.

### Statistical analysis

The Student’s *t-*test and One-way ANOVA (Dunnett’s test) were used to determine the statistical differences between various experimental and control groups. A *p* value of < 0.05 was considered significant.

## Additional Information

**How to cite this article**: Kue, C. S. *et al*. Tropomyosin Receptor Kinase C Targeted Delivery of a Peptidomimetic Ligand-Photosensitizer Conjugate Induces Antitumor Immune Responses Following Photodynamic Therapy. *Sci. Rep.* **6**, 37209; doi: 10.1038/srep37209 (2016).

**Publisher’s note**: Springer Nature remains neutral with regard to jurisdictional claims in published maps and institutional affiliations.

## Supplementary Material

Supplementary Information

## Figures and Tables

**Figure 1:**
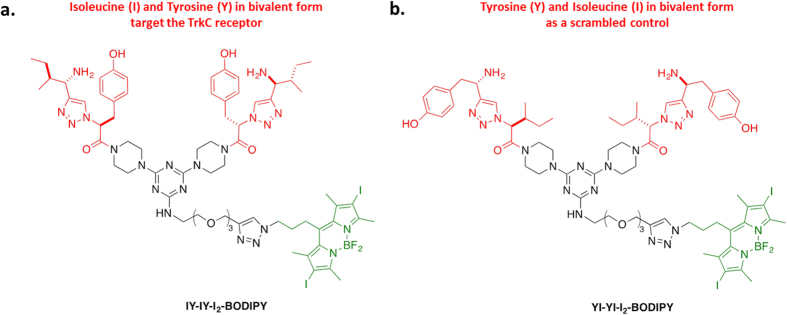
Structures of the designed conjugates featured in this work. (**a**) IYIY-I_2_-BODIPY is TrkC receptor targeted conjugate and (**b**) YIYI-I_2_-BODIPY is a scrambled control. Red represents the designed peptide-ligand portion, black for the linker and green for the parent iodinated BODIPY (I_2_-BODIPY).
