# Supplementary material for: Tropomyosin Receptor Kinase C Targeted Delivery of a Peptidomimetic Ligand-Photosensitizer Conjugate Induces Antitumor Immune Responses Following Photodynamic Therapy
Source: Sci Rep. 2016 Nov 17;6:37209. doi: 10.1038/srep37209 (PMC5112560; doi:10.1038/srep37209)
Supplement: Supplementary Information [file srep37209-s1.pdf]

Tropomyosin receptor kinase C Targeted Delivery of a Peptidomimetic Ligand-Photosensitizer Conjugate Induces Antitumor Immune Responses Following Photodynamic Therapy

Chin Siang KUE[1*], Anyanee KAMKAEW[2], Siew Hui VOON[1], Lik Voon KIEW[1*], Lip Yong CHUNG[3], Kevin BURGESS[2], Hong Boon LEE[3*]

1. Department of Pharmacology, Faculty of Medicine, University of Malaya, 50603 Kuala Lumpur, Malaysia.
2. Department of Chemistry, Texas A & M University, Box 30012, College Station, Texas 77842, United States.
3. Department of Pharmacy, Faculty of Medicine, University of Malaya, 50603 Kuala Lumpur, Malaysia.

Correspondence should be addressed to

**LEE Hong Boon (main);* Email*:* hongboonlee@um.edu.my; Phone: +603-79674971

**KUE Chin Siang;* Email: cskue85@yahoo.com

**KIEW Lik Voon;* Email: lvkiew@ummc.edu.my

Short title: Antitumor immunity of TrkC targeted PDT conjugates

Keywords:

Tropomyosin receptor kinase C (TrkC), diiodo-boron dipyrromethene (I_2-BODIPY), antitumor immunity, active targeting, Photodynamic therapy (PDT).

Supplementary Figures

Supplementary Figure 1

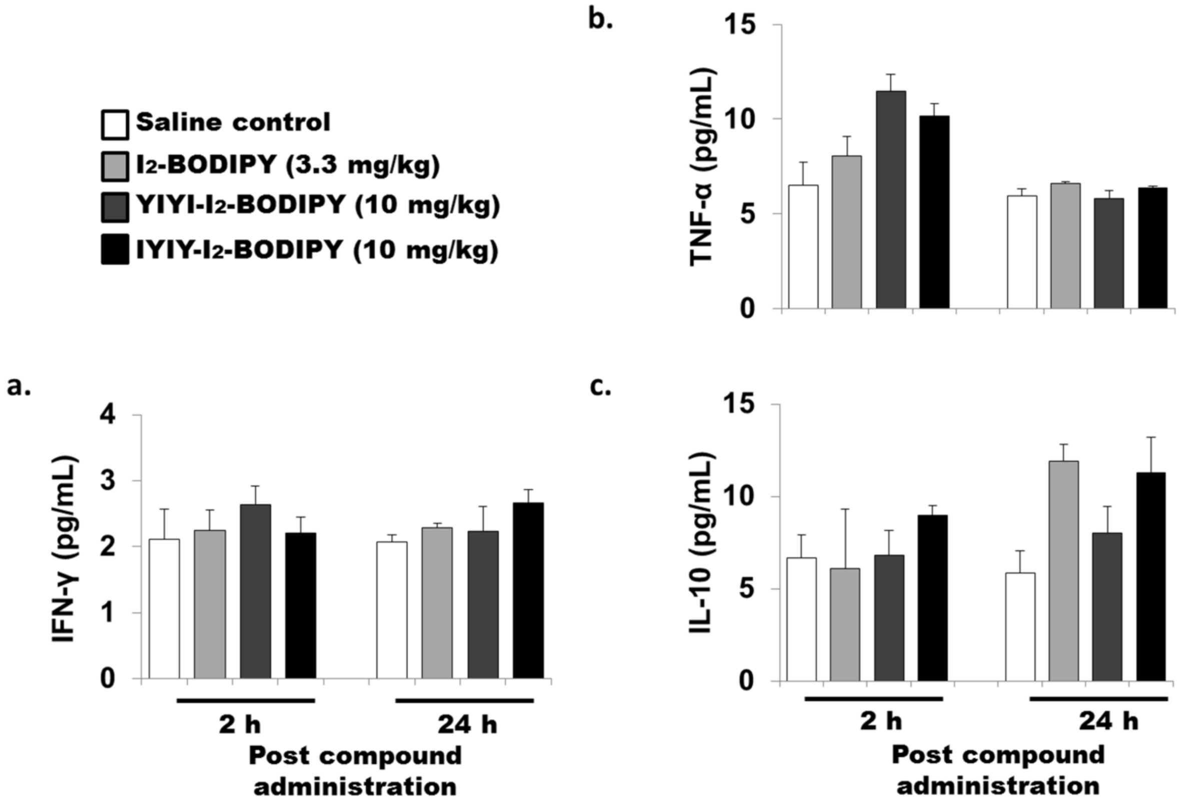

Supplementary Figure 1: Cytokines profile in plasma of compounds treated mice in dark. 4T1 tumor bearing mice were randomly divided into four treatment groups (saline, I_2-BODIPY, YIYI-I_2-BODIPY and IYIY-I_2-BODIPY) and compounds were administrated via tail vein respectively. Mice were then sacrificed at 2 h and 24 h post compounds administration. The blood plasma was extracted from these mice via cardiac puncture for cytokines analysis. Cytokines (**a**) IFN-γ, (**b**) TNF-α and (**c**) IL-10 are shown. Data represent mean ± SEM with minimum of four mice per group.

Supplementary Figure 2

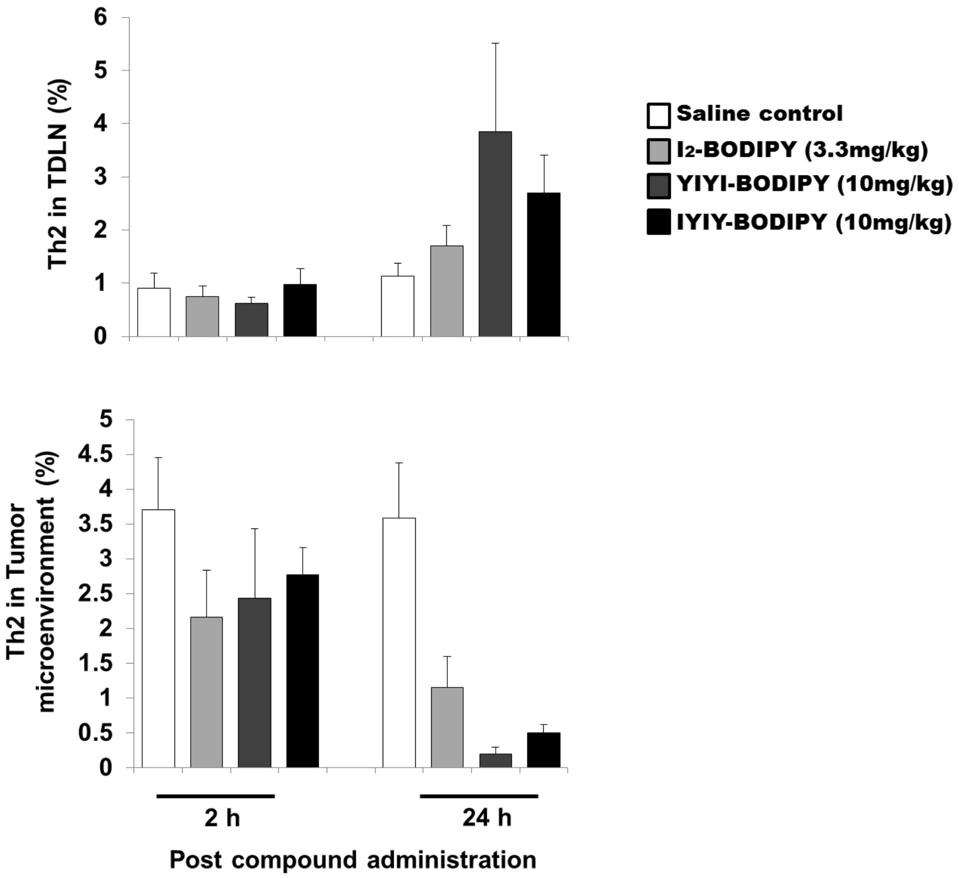

Supplementary Figure 2: T helper 2 cell populations at TDLN and TM in dark condition. 4T1 tumor bearing mice were treated with respective compounds via tail vain. For dark (non-irradiation), mice were kept in dark and sacrificed at indicated time. Th2 cells in (a) TDLN and (b) tumor microenvironment were quantified using flow cytometry. Data represent mean ± SEM of minimum four mice in each group.

Supplementary Figure 3

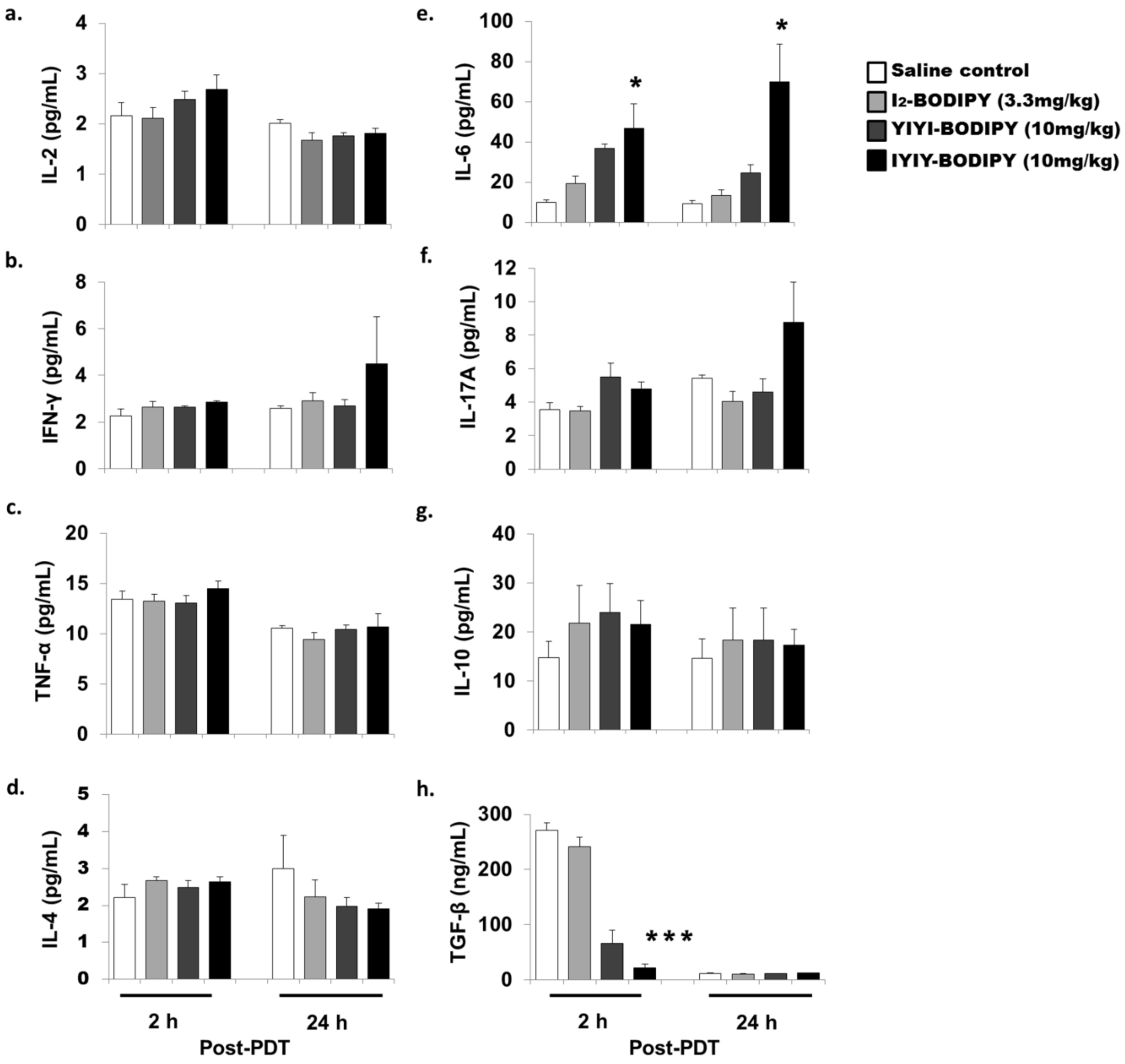

Supplementary Figure 3: Compounds-modulated cytokines changes following irradiation. 4T1 tumor bearing mice were treated with respective compounds via tail vain. Mice were kept in dark for 1 h, anaesthetized and irradiated with 100 J/cm^2 of light at a fluence rate of 160 mW/cm^2. Mice were then sacrificed at 2 h and 24 h post-PDT. The blood plasma was extracted from these mice via cardiac puncture for cytokines analysis. Cytokines (**a**) IL-2, (**b**) IFN-γ, (**c**) TNF-α, (**d**) IL-4, (**e**) IL-6, (**f**) IL-17A, (**g**) IL-10 and (**h**) TGF-β are shown. Data represent mean ± SEM with minimum of four mice per group. * $p < 0.05$, *** $p < 0.001$ *vs* I_2-BODIPY using one-way ANOVA (Dunnett's test).

Supplementary Figure 4

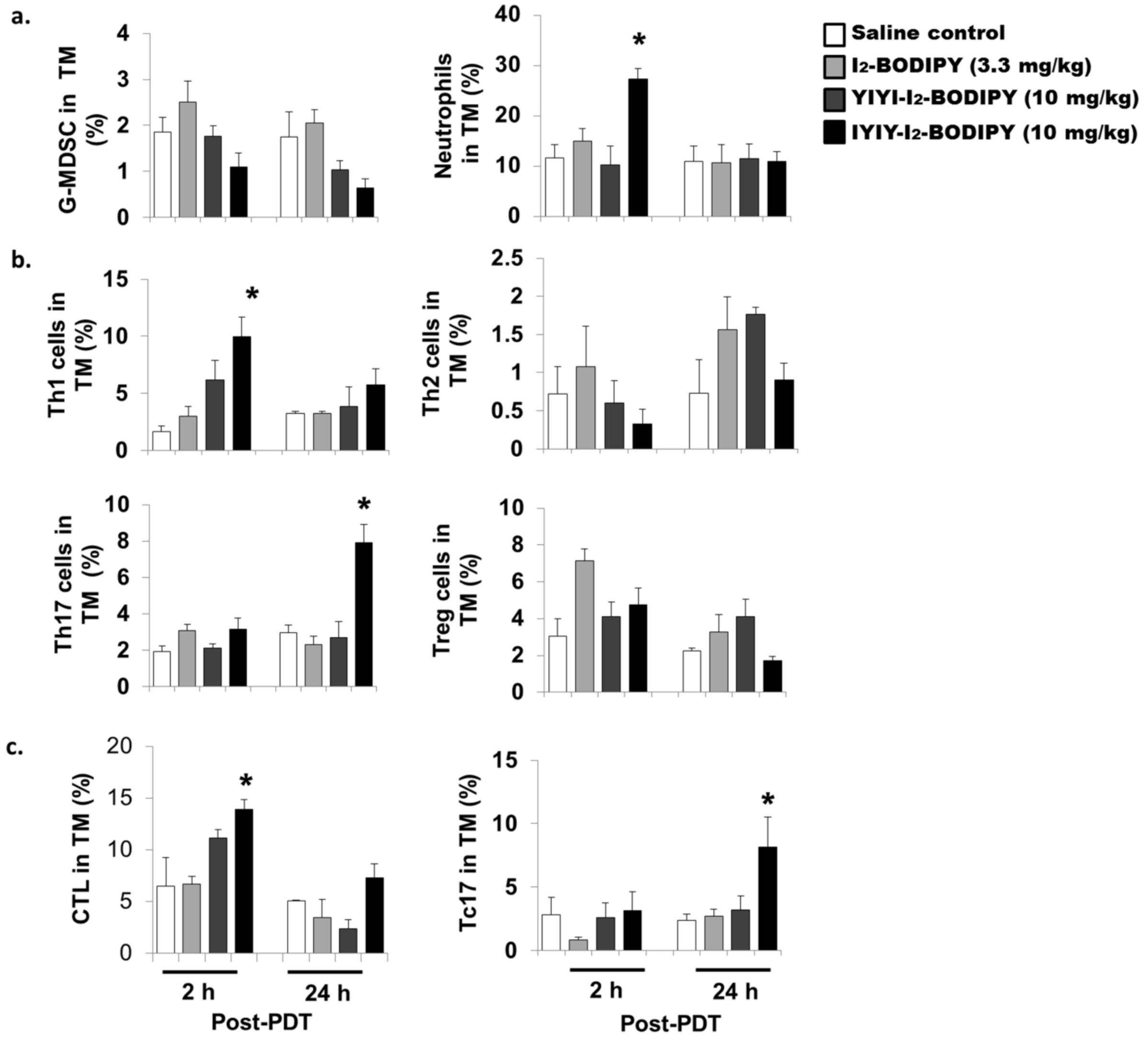

Supplementary Figure 4: Compounds-modulated immune cells populations following irradiation. 4T1 tumor bearing mice were treated with respective compounds via tail vain. Mice were kept in dark for 1 h, anaesthetized and irradiated with 100 J/cm^2 of light at a fluence rate of 160 mW/cm^2. Mice were then sacrificed at 2 h and 24 h post-PDT. Suspension cells were obtained from tumor microenvironment and quantified based on the methods explained in M&M. Data of (**a**) myeloid cells (G-MDSCs, neutrophils) (**b**) CD4+ T helper cells (Th1, Th2, Th17, Treg), (**c**) CD8+ T cells (CTL, Tc17) quantification using flow cytometry. Data represent mean ± SEM of minimum four mice in each group. * $p < 0.05$, *vs* I_2-BODIPY using one-way ANOVA.
